# A novel non-Hodgkin lymphoma murine model closer to the standard clinical scenario

**DOI:** 10.1186/s12967-016-1073-8

**Published:** 2016-11-22

**Authors:** Thais Bascuas, María Moreno, Amy Mónaco, Laura Reyes, Andrea Paolino, Patricia Oliver, María G. Kramer, Henry Engler, José P. Pacheco, Sofía Grille, José A. Chabalgoity

**Affiliations:** 1 Departamento de Desarrollo Biotecnológico, Instituto de Higiene, Facultad de Medicina, UdelaR, Montevideo, Uruguay; 2 Cátedra de Hematología, Hospital de Clínicas, Facultad de Medicina, UdelaR, Montevideo, Uruguay; 3 Área de Investigación y Desarrollo, Departamento Biomédico, Centro Uruguayo de Imagenología Molecular (CUDIM), Montevideo, Uruguay; 4 Área de Patología Funcional y Morfológica, Departamento de Patología, Facultad de Veterinaria, UdelaR, Montevideo, Uruguay

**Keywords:** Non-Hodgkin lymphoma (NHL), Immunotherapy, Chemotherapy, CHOP, Clinical, Positron emission tomography (PET), Minimal residual disease (MRD)

## Abstract

**Background:**

Non-Hodgkin lymphomas (NHL) are the most frequent hemato-oncological malignancies. Despite recent major advances in treatment, a substantial proportion of patients relapses highlighting the need for new therapeutic modalities. Promissory results obtained in pre-clinical studies are usually not translated when moving into clinical trials. Pre-clinical studies are mainly conducted in animals with high tumor burden; instead patients undergo chemotherapy as first line of treatment and most likely are under remission when immunotherapies are applied. Thus, an animal model that more closely resembles patients’ conditions would be a valuable tool.

**Methods:**

BALB/c mice were injected subcutaneously with A20 lymphoma cells and after tumor development different doses of chemotherapy were assessed to find optimal conditions for minimal residual disease (MRD) establishment. Tumor growth and survival, as well as drugs side effects, were all evaluated. Complete lymphoma remission was monitored in vivo using positron emission tomography (PET), and the results were correlated with histology. Immunological status was assessed by splenocytes proliferation assays in NHL-complete remission mice and by analyzing tumor cell infiltrates and chemokines/cytokines gene expression in the tumor microenvironment of animals with residual lymphoma.

**Results:**

Two cycles of CHOP chemotherapy at days 25 and 35 post-tumor implantation induced complete remission for around 20 days. PET showed to be a suitable follow-up technique for MRD condition with 85.7 and 75% of sensibility and specificity respectively. Proliferative responses upon mitogen stimulation were similar in animals that received chemotherapy and wild type mice. Tumors from animals with residual lymphoma showed higher numbers of CD4^+^ and CD8^+^ and similar numbers of NK, neutrophils and Tregs infiltrating cells as compared with non-treated animals. Gene expression of several cytokines as well as an array of chemokines associated with migration of activated T cells to tumor sites was upregulated in the tumor microenvironment of animals that received chemotherapy treatment.

**Conclusions:**

We established a NHL-B pre-clinical model using standard chemotherapy to achieve MRD in immunocompetent animals. The MRD condition is maintained for approximately 20 days providing a therapeutic window of time where new immunotherapies can be tested in conditions closer to the clinics.

## Background

Non-Hodgkin lymphomas (NHL) are the most frequent hemato-oncological malignancies. The current standard of care for NHL treatment is chemotherapy, radiotherapy and monoclonal antibodies (i.e. Rituximab) [1]. Although these combined treatments can achieve high rates of complete remission, a substantial proportion of patients relapses with chemoresistant disease. Survival rates for NHL vary depending on the age, performance status, histological type, stage, (international prognostic index) IPI score and others prognostic factors. According to SEER Cancer Statistics Review (CSR), the overall 5-year relative survival rate for all ages is 69%, and the 10-year relative survival rate is 59%; 86% for follicular lymphoma, and 61% for diffuse large B-cell lymphoma (DLBCL) [2].

Active immunotherapy is a promising complementary approach for NHL treatment. Induction of tumor-specific adaptive immunity might generate long-lasting immunological memory able to prevent further relapses, and there is abundant literature demonstrating good rates of effectivity in pre-clinical models [3–5]. However, overall 85% of phase I–II clinical trials for novel treatments in cancer fail; and of those that progress to phase III, only half become approved for clinical use [6]. Pre-clinical NHL active immunotherapy studies are mainly performed in mice with high tumor burden that received no previous treatment, whereas clinical trials are performed in patients that have undergone chemotherapy and they are in complete or partial remission [3, 7]. Thus, murine models usually do not resemble the clinical scenario and this can be a major reason to explain the lack of consistency in results when moving into clinical trials [8]. The development of novel pre-clinical models that mimic the clinical setting could be valuable tool to explore new therapeutic strategies [9]. Particularly, it would be important to have a model in which animals achieved complete remission with chemotherapy and develop minimal residual disease (MRD) state.

We have previously assessed a murine NHL-B model where different immunotherapies were evaluated with promising results [10–12]. In the present study, we establish a new NHL-B pre-clinical model using standard chemotherapy (CHOP) to achieve complete response in immunocompetent animals. We believe that this model is more suitable to study new and optimized immunotherapies, thus it could be a valuable tool for pre-clinical studies that get closer to the clinics.

## Methods

### Animals and tumor cell line

Female BALB/c mice, 8–10 weeks old were used for in vivo experiments. Animals were housed on a 12:12 h light/dark cycles in racks with filtered air where food and water were given ad libitum.

The A20 cell line was derived from B lymphocytes of a naturally occurring reticulum cell sarcoma from an old BALB/cAnN mouse and was obtained from the American Type Culture Collection (Manassas, VA, USA). Cells were grown in RPMI-1640 medium (Sigma-Aldrich, St. Louis, MO) supplemented with 10% fetal bovine serum (PAN-Biotech, Aidenbach, Germany), 2 mM l-glutamine (Sigma) and 0.05 mM 2-mercaptoethanol (Sigma-Aldrich, St. Louis, MO) (RPMI complete medium) at 37 °C in 5% CO_2_ atmosphere.

### Cell transplantation

For tumor challenge, A20 were grown in culture and harvested in log phase, washed and resuspended to a final concentration of 5 × 10^6^ cells/ml in PBS. 8–10 weeks-old syngeneic female BALB/c mice were injected subcutaneously (s.c.) into the right flank with 1 × 10^6^ cells in 0.2 ml of PBS. Tumors were measured three times a week with a microcaliper in three dimensions, and tumor volumes were calculated as length × width × depth × 0.5236, as previously described [13]. Mice were euthanized by cervical dislocation when tumors reached 3000 mm^3^ or before if they showed signs of distress. These time points were defined as survival time.

### Chemotherapy treatment for MRD lymphoma model

The standard chemotherapy regimen in patients for aggressive NHL as well as the most used regimen for indolent NHL is a combination of cyclophosphamide, doxorubicin, vincristine and prednisone/steroids (CHOP) given every 21 days for 6–8 cycles [1, 14]. We used a CHOP regimen to treat mice bearing lymphoma where oral prednisone was substituted by intraperitoneal (i.p.) dexamethasone in order to achieve more uniform levels of steroid and to reduce chemical peritonitis. In each chemotherapy cycle we used the following doses: cyclophosphamide 100 mg/kg i.p, doxorubicin 6 mg/kg i.p, vincristine 0.1 mg/kg i.p and dexamethasone 0.2 mg/kg i.p. At day 25 post-tumor implantation (p.t.i.), groups of mice (n = 9) that were inoculated with A20 cell line were treated either with one cycle of chemotherapy (CHOP×1), two cycles of chemotherapy (CHOP×2) or PBS as control and were followed for tumor growth and survival. Side effects were monitored, through weight changes evaluation and hematological toxicity. Lymphocytes, monocytes and neutrophils recovery post-CHOP were evaluated in an automated blood cell counter and in peripheral blood smears. Blood was obtained using a 27G needle puncturing the tail vein. This allows us to select the optimal period between CHOP cycles.

To avoid potential infections, prophylactic anti-infectious drugs (15 mg/kg of fluconazole and 20 mg/kg of acyclovir) were used during the neutropenia period.

### Chemotherapy cytotoxicity

Cytotoxic effect of CHOP drugs on A20 tumor cell line was assessed in vitro by growth inhibition studies. Cells were seeded in 24-well plates at a density of 1 × 10^6^ viable cells per well. Triplicate wells were treated with different concentrations of the drugs or PBS and Dimethyl sulfoxide (DMSO) as controls (Sigma-Aldrich, St. Louis, MO). Cyclophosphamide, doxorubicin, and dexamethasone were tested 0.1, 1, 10, 100 nM and 1 µM, and Vincristine was tested 10, 20, 30, 40, 50 and 70 nM. Plates were incubated at 37 °C in a humidified incubator with 5% CO_2_ for 96 h. Then, all cultures were monitored for cell viability using Trypan blue vital stain (Life Technologies, Carlsbad, CA), and Annexin V (BD Pharmingen, San Diego, CA) with Propidium iodide (PI) (Sigma-Aldrich, St. Louis, MO). The half maximal inhibitory concentration (IC_50_) was calculated as the concentration (nM) that causes half of the cell mortality.

To evaluate in vivo toxicity, body weight was measured before and after CHOP administration. The body weight change (BWC) was calculated using the following formula: ${\text{BWC }}\left( \% \right) = \left[ {\left( {\text{body weight on the last day}} \right){-}\left( {{\text{body weight on day }}0} \right)} \right]/\left( {{\text{body weight on day }}0} \right) \times 100 \, \left( \% \right)$, as previously described [15].

### Necropsy and histology

Mice were sacrificed at day 45 p.t.i. and necropsies were performed. Animals were examined macroscopically for lymphoma infiltration at primary sites or lymph nodes or other organs. Primary lymphoma site, inguinal and retroperitoneal lymph nodes, liver and spleen were removed and immersed in formalin buffered 10% at room temperature for 24 h. Organs were washed with PBS for 24 h at 4 °C and then immersed successively two times for 2 h in EtOH 95%, absolute alcohol, Xylol, paraffin and processed in paraffin blocks. Sections of 4 µm were cut and dewaxing immersed successively two times for 5 min in xylol and absolute alcohol, one time for 5 min in EtOH 95% and EtOH 90%. Then sections were washed three times with distilled water and stained with hematoxylin-eosin. Finally, sections were immersed successively for 3 s in EtOH 70%, EtOH 80%, EtOH 95%, absolute alcohol and Xylol, and assembled with Canada balsam. These sections were microscopically evaluated to detect lymphoma infiltration.

### PET/CT imaging

Complete lymphoma remission was evaluated by in vivo imaging using positron emission tomography/computed tomography (PET/CT) scan with ^18^F-2-fluor-2-desoxi-d-glucosa (^18^F-FDG) as radiotracer. The study was performed in the Centro Uruguayo de Imagenología Molecular (CUDIM).

#### Image acquisition

PET/CT imaging in mice was performed using a tri-modality scanner (Triumph, Trifoil, Inc., US) with LYSO/LGSO scintillators (spatial resolution: 1.0 mm and an axial field of view: 3.75 cm). Data were acquired in list mode in a 184 × 184 × 31 matrix with pixel size: 0.25 × 0.25 × 1.175 mm; coincidence window width: 22.22 s.

Seventeen mice were evaluated by PET/CT, of which two died later on as result of cytotoxicity and thus were excluded from the analysis. Images were obtained before (day 25 p.t.i.) and after CHOP treatment when mice were in complete clinical remission (day 44 p.t.i.). The animals were anesthetized with 2% isofluorane in an oxygen flow of 2 l/min, placed in prone position on the scanner bed and injected via the caudal vein with 100 µl of (^18^F-FDG) (20 ± 5.4 MBq). PET images (static studies) acquisition started 30 min after radiotracer administration and performed over 60 min. Sinograms were reconstructed using 3D maximum likelihood expectation maximization (3D-MLEM).

#### Image analysis

Semi-quantitative analysis was done using PMOD software version 3.4 (PMOD Technologies, Ltd., Zurich, Switzerland). PET studies were co-registered with the corresponding CT scan studies for anatomical localization. Images were displayed as coronal, sagittal and axial slices.

For quantifying the specific uptake, volumes of interest (VOIs) were drawn manually on tumor as target (T) and skeletal muscle as non-target tissue (NT) [16]. The activity uptake was expressed as KBq/cc; results were expressed as T/NT.

### Splenocyte proliferation assay

At day 45 p.t.i. mice in complete remission from CHOP×2 group as well as mice from PBS control were sacrificed and spleens were removed. Spleens were disrupted and prepared as a single-cell suspension. Splenocytes were stained with 1 µM of 5(6)-carboxyfluorescein diacetate *N*-succinimidyl ester (CFSE) (Sigma-Aldrich, St. Louis, MO). CFSE labeled splenocytes were incubated at 37 °C with 5% CO_2_ in 24 wells plate at 2 × 10^6^ cells/ml density by triplicates in complete medium for 4 days and were stimulated with 1 µg/ml of Concanavalin A (ConA) (Sigma-Aldrich, St. Louis, MO) or non-stimulated. After the incubation, cells were washed and stained with PI for 10 min in the dark at room temperature. Data were acquired by flow cytometry (FACS Canto II, Becton–Dickinson) and were analyzed with ModFit software (Verity Software House, Inc). For analysis, viable cells were gated based on FSC and PI profiles. The parent generation was set as the median fluorescence intensity using non-stimulated control sample.

### Flow cytometry analysis of tumor-infiltrating cells

At day 45 p.t.i. mice with residual primary lymphoma from CHOP×2 group (i.e. animals with partial response to chemotherapy) as well as PBS control, were sacrificed and tumors were removed and prepared as a single-cell suspension. Cells were immunostained at 4 °C in the dark for 30 min with the following panel of antibodies: FITC-conjugated anti-CD49b, PECy7-conjugated anti-CD8, APC-conjugated anti-CD3, APCCy7-conjugated anti-CD4, PerCPCy5.5-conjugated anti-CD19, FITC-conjugated anti-CD4, PE-conjugated anti-FoxP3, PECy7-conjugated anti-CD3, APC-conjugated anti-CD25, FITC-conjugated anti-Ly6C, PE-conjugated anti-Ly6G, (all reagents from BD Pharmingen, San Diego, CA). Optimal antibody concentration was previously defined by titration. For intracellular FoxP3 staining, cells were first stained with anti-CD4 and anti-CD25 antibodies, then fixed and permeabilized with a mouse FoxP3 buffer set (BD Pharmingen) according to the manufacturer’s protocols. Cells were washed twice with permeabilization buffer and then incubated with anti-FoxP3 at 4 °C for 30 min in the dark. Flow cytometry data were collected on a FACS Canto II flow cytometer equipped with two lasers (Becton–Dickinson, Oxford, UK). For data acquisition and analysis, FACSDiva (Becton–Dickinson) and Infinicyt (Cytognos, Spain) software were used.

### Determination of cytokines and chemokines in tumor microenvironment

Tumor microenvironment cytokines and chemokines mRNA levels were determined using quantitative reverse transcription-PCR. At day 45 p.t.i mice from PBS and CHOPx2 group, in which primary tumor persisted (partial remission), were sacrificed and tumors were removed, immersed in Trizol reagent (Invitrogen, Carlsbad, CA) and stored at −80 °C until processed. Tumors were homogenized with an Ultra Turrax T8 homogenizer (IKA-Werke, Staufen, Germany) and RNA was extracted according to the manufacturer’s instructions. RNA quality and quantity were assessed by spectrophotometric measurements at 260/280 nm in a NanoDrop 2000 (Thermo Fisher Scientific, Waltham, MA). Before cDNA synthesis, 1 µg total RNA was treated with DNase I (Invitrogen), and first-strand cDNA synthesis was carried out using random primers (Invitrogen) and Moloney murine leukaemia virus reverse transcriptase (Invitrogen). Real-Time PCR (RT-PCR) was performed using a QuantiTect SYBR green PCR kit (Qiagen, Hilden, Germany) in a 7900HT RT-PCR System (Applied Bio- systems, Foster City, CA). *B2m* gene was used as housekeeping gene. The primers used are listed in Table 1 and were used at a final concentration of 0.9 µM. The relative mRNA amount in each sample was calculated using the 2^−ΔΔCt^ method [17], where $\Delta {\text{Ct }} = {\text{ Ct}}_{\text{gene of interest}} {-}{\text{ Ct}}_{B2m}$, and expressed as relative mRNA levels in the test group compared with the control group (fold change).

**Table 1:** Sequences of primers used for quantitative RT-PCR

Gene	Forward primer (5′–3′)	Reverse primer (5′–3′)	Product length (bp)
*B2m*	CCTGCAGAGTTAAGCATGCCAG	TGCTTGATCACATGTCTCGATCC	72
*Ccl2*	CCCTCAACGGAAGAACCAAA	CACATCAGGTACGATCCAGGC	72
*Ccl3*	AACATCATGAAGGTCTCCAC	CCAAGACTCTCAGGCATTCA	294
*Ccl4*	GCCCTCTCTCTCCTCTTGCT	GTCTGCCTCTTTTGGTCAGG	196
*Ccl5*	GGTACCATGAAGATCTCTGCA	AAACCCTCTATCCTAGCTCAT	294
*Ccl20*	TTTTGGGATGGAATTGGACAC	TGCAGGTGAAGCCTTCAACC	69
*Cxcr4*	TTCTCATCCTGGCCTTCATC	CTTTTCAGCCAGCAGTTTCC	92
*Cxcr7*	GCCGTACCATTTTGTGGTTC	TGCAACGCTGTAAAGAGCAC	96
*Cxcl1*	CTTGGTTCAGAAAATTGTCCAAAA	ACGGTGCCATCAGAGCAGTCT	84
*Cxcl9*	TGGAGCAGTGTGGAGTTCGA	CCTCGGCTGGTGCTGATG	73
*Cxcl10*	GCCGTCATTTTCTGCCTCAT	GCTTCCCTATGGCCCTCATT	127
*Cxcl11*	CAAAATGGCAGAGATCGAGAAA	TGAGCCTTCATAGTAACAATCACTTCA	87
*Cxcl12*	GAAGTGGAGCCATAGTAATGCC	TCCAAGTGGAAAAATACACCG	133
*Cxcl13*	CAACTGTTGTCGGTCTAAACATCAT	GGTCCAGATCACAACTTCAGTTTTG	89
*Gal*-*1* (Galectina-1)	TGAACCTGGGAAAAGACAGC	TCAGCCTGGTCAAAGGTGAT	190
*Il2*	CCTGAGCAGGATGGAGAATTACA	CTTTCAATTCTGTGGCCTGCTTGGG	92
*Il4*	ACAGGAGAAGGGACGCCAT	GAAGCCCTACAGACGAGCTCA	95
*Il6*	GTTCTCTGGGAAATCGTGGAAA	AAGTGCATCATCGTTGTTCATACA	78
*Il10*	CATTTGAATTCCCTGGGTGAGA	TGCTCCACTGCCTTGCTCTT	101
*Il12*	ATCACACGGGACCAAACCA	CAGGCAACTCTCGTTCTTGTGTAGT	74
*Il17a*	CTCCAGAAGGCCCTCAGACTAC	GGGTCTTCATTGCGGTGG	69
*Ifng*	TCAGCAACAGCAAGGCGAAA	CCGCTTCCTGAGGCTGGAT	143
*Tgfb*	GCTGAACCAAGGAGACGGAAT	GAGTTTGTTATCTTTGCTGTCACAAGA	76
*Foxp3*	CCCAGGAAAGACAGCAACCTT	TTCTCACAACCAGGCCACTTG	89
*Tnfa*	CATCTTCTCAAAATTCGAGTGACAA	CCTCCACTTGGTGGTTTGCT	63

### Statistical analysis

Results were analyzed in SPSS 17.0 (Statistical Package for the Social Sciences) for Windows. Differences in survival times were determined using Kaplan–Meier and log-rank test. For tumor growth and in vitro assays the statistical significance of differences between study groups were analyzed using Student’s t-test and analysis of variance (ANOVA). A value of p < 0.05 was considered statistically significant.

Receiver operating characteristic curve (ROC) was used to evaluate the T/NT ratio in the diagnosis of complete remission by assessing the area under the ROC curve (AUC), sensitivity, and specificity. The cut-off point was determined by maximizing the sum of sensitivity and specificity; histology was used as a gold standard variable.

## Results

### Chemotherapy efficacy and in vivo toxicity

First, we assessed the bioactivity of the cytostatic drugs on the A20 tumor cell line. The in vitro IC_50_ values obtained for doxorubicin and vincristine were 25 and 1 nM respectively, which are similar to the reported IC_50_ values of these two drugs on other tumor-sensitive cell lines, confirming the possibility of using them on the lymphoma model. Our results also confirmed that cyclophosphamide and dexamethasone cannot be evaluated in vitro since they both requires hepatic activation [18].

**Fig. 1:**
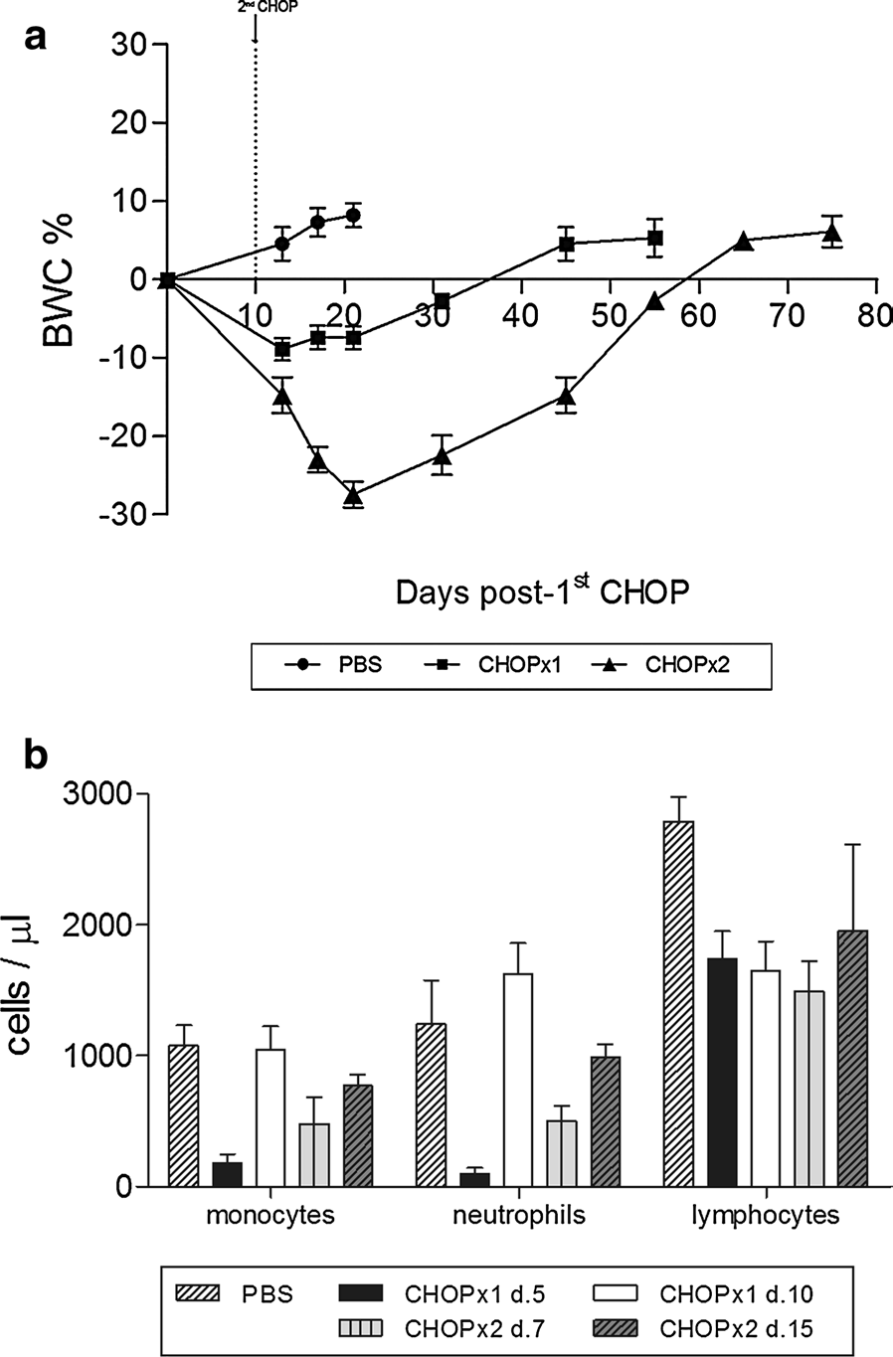
In vivo CHOP toxicity. **a** Percentage of body weight change (BWC) is shown for PBS, CHOP×1 and CHOP×2 groups. **b** Peripheral white blood cells counts. Absolute values for monocytes, neutrophils and lymphocytes are shown for untreated (PBS group), treated animals, 5 and 10 days post- first CHOP cycle (CHOP×1 d.5 and CHOP×1 d.10, respectively) and 7 and 15 days post- second CHOP cycle (CHOP×2 d.7 and CHOP×2 d.15, respectively)

We then treated A20-bearing animals with increasing doses of CHOP starting with doses used in a previously reported xenograft lymphoma model [19]. Those particular doses did not show any anti-tumor effect on A20-bearing immunocompetent animals, so we doubled them and found that they were too toxic and all animals succumbed to the treatment (results not shown). Thus, we decided to assess intermediate values and to apply two cycles separated ten days apart. This interval between cycles was decided based on results from white blood cells peripheral count that demonstrated complete bone marrow regeneration at day 10 post-chemotherapy. Overall, these studies provided us with an optimal CHOP doses regime for treatment as described in Methods. At these particular doses, animals had an acceptable tolerance to chemotherapy. Particularly, no animals died with one cycle of CHOP, while 18% of animals receiving two cycles of CHOP died by chemotherapy cytotoxicity. Low levels of toxicity were also evidenced by variations in body weight over time (Fig. 1a). The highest decrease in weight was observed ten days after second CHOP cycle, and after that, animals started to recover towards average normal body weight. Cachexia was not observed at any time after CHOP administration. Transient neutropenia and monocytopenia were observed post CHOP, but normal values were recovered by day 10–15. Both CHOP cycles induced transient myelosuppression (Fig. 1b). Instead, lymphocytes levels in blood also decreased after CHOP, and values did not return to previous values within the time frame of the experiments.

All in all, these results showed that we had found a suitable chemotherapy treatment with reasonable toxicity levels.

### Tumor development and survival rate

**Fig. 2:**
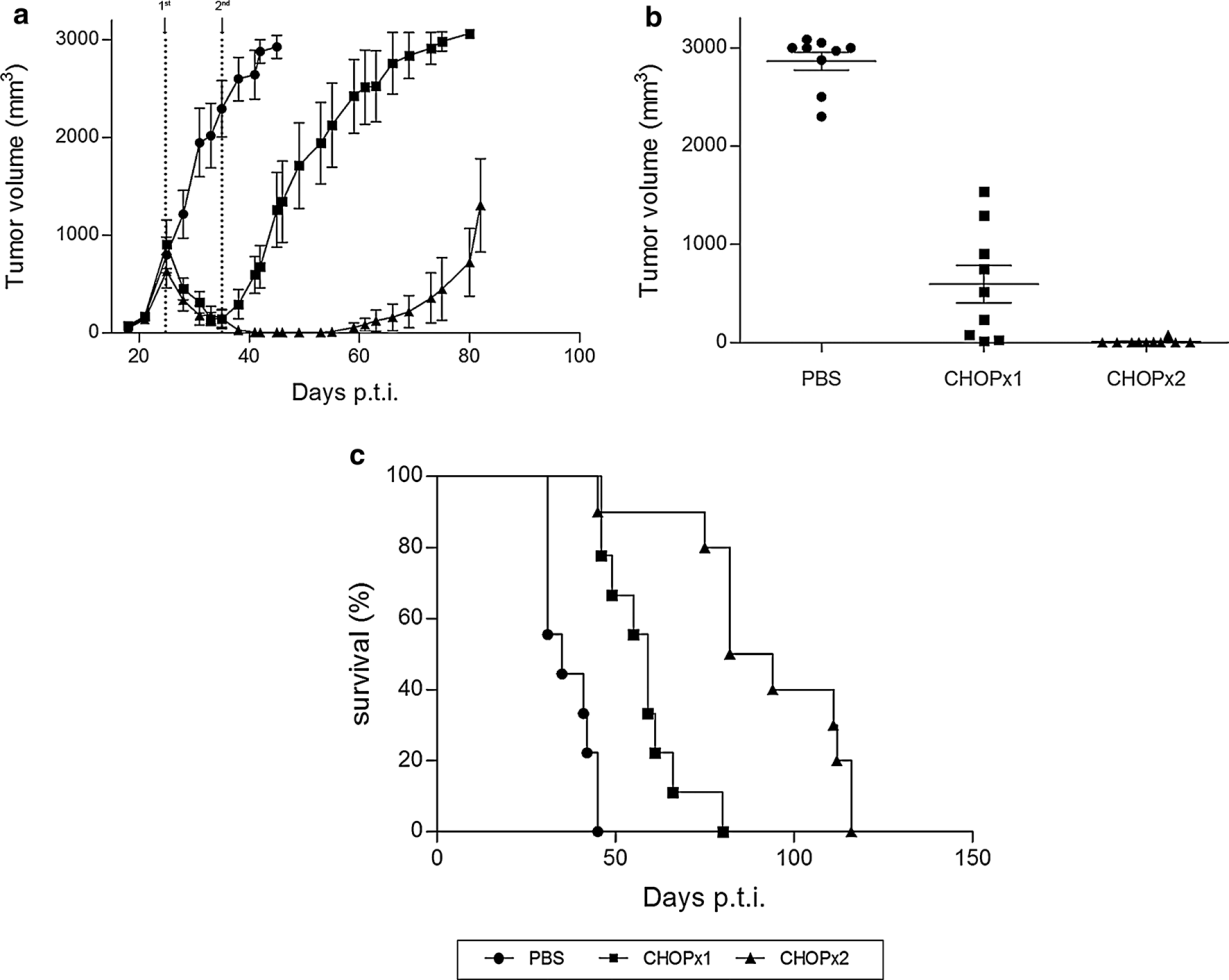
Tumor growth and animal survival in MRD model. **a** Tumor volume (mm^3^) was measured every 2–3 days. Results are shown as mean ± standard deviation (n = 9). CHOP cycles are indicated with *arrows*. **b** Tumor volume (mm^3^) at day 41 p.t.i. Each *dot* represents one individual animal. Mean and standard deviation are also depicted. Significant differences were observed between all groups (p < 0.0001). **c** Animal survival was followed up for 120 days. Significant differences were observed between all groups (log rank, p < 0.0001)

We then evaluated the effect of the selected CHOP regimen on tumor development and survival. As previously shown by us [10], A20-bearing animals that do not receive any treatment show sustained tumor growth over time and a median survival of around 35 days. On the contrary, animals receiving either one or two doses of chemotherapy showed a decrease in tumor growth from day 25 p.t.i., starting immediately after the first CHOP cycle (Fig. 2a). Mice receiving a single CHOP dose had a period when tumor volume decreases and it was close to zero, but this status lasted only for a few days. Instead, in the group of animals receiving two doses of CHOP, around 92% of animals showed clinical complete remission of the primary tumor for a longer period of approximately 20 days (Fig. 2a, b). After that tumors started to grow again and eventually all mice died for lymphoma disease. Reasons of death among the animals depended of the particular treatment group and were either drug toxicity or respiratory distress, most likely due to metastasis, or the animals where euthanized for ethical reasons when the tumor reached a pre-defined volume size of 3000 mm^3^ (Table 2). Survival among animals receiving any chemotherapy regimen was extended, but it was significantly higher among animals receiving two cycles of chemotherapy (Fig. 2c).

**Table 2:** Percentage of animal death related to drug resistance, metastatic disease and primary tumor

	PBS	CHOP×1	CHOP×2
Drug toxicity	NA	0%	18%
Metastatic disease	0%	25%	47%
Primary tumor (>3000 mm^3^)	100%	75%	35%

*NA* not applicable

### Evaluation of remission in treated animals

#### Histology

**Fig. 3:**
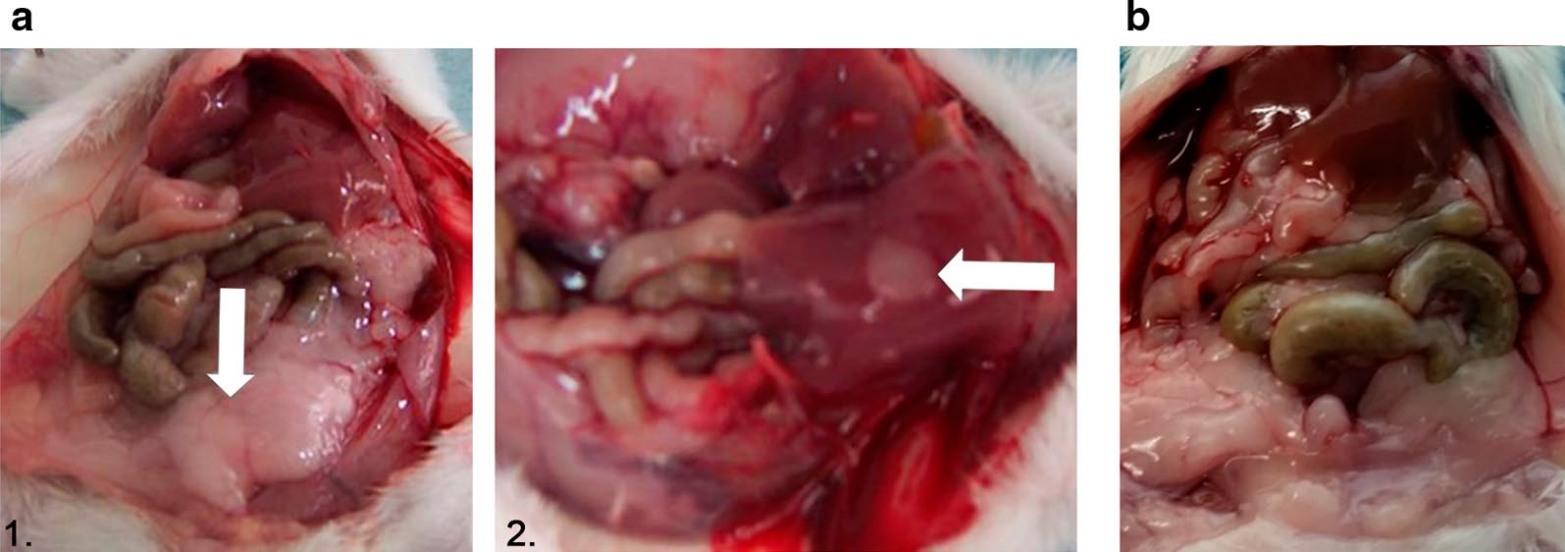
Necropsy of chemotherapy-treated and untreated animals at day 45 p.t.i. **a** Untreated mouse: retroperitoneal (*1*) and hepatic (*2*) involvement are indicated with* arrows*. **b** CHOP×2-treated mouse in complete remission

**Fig. 4:**
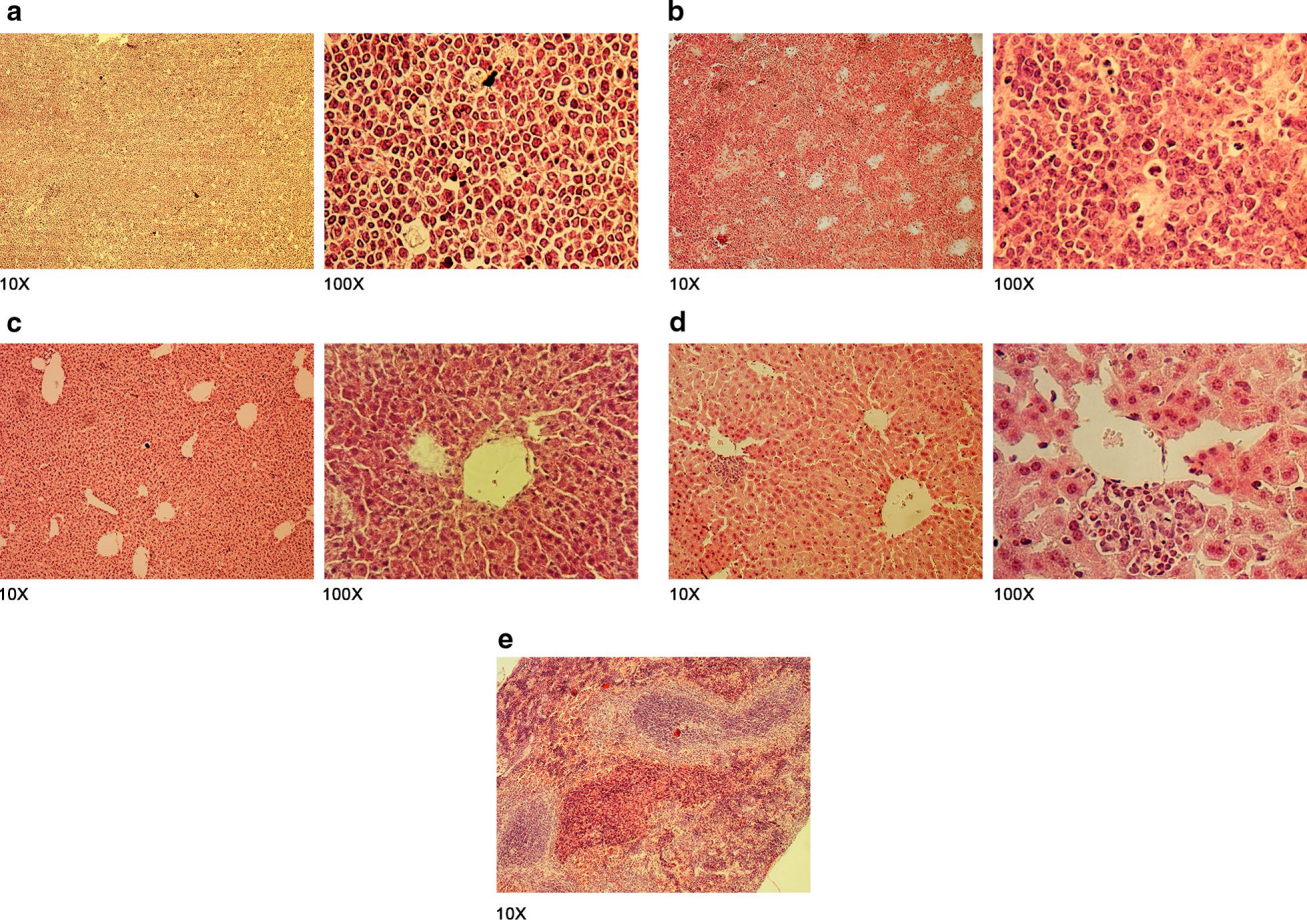
Histological study of MRD. **a** Primary tumor sections from a control mouse (PBS group): NHL-B histology is observed and corresponds to a diffuse infiltration by centrocytes and centroblasts with a starry sky pattern, due to high proliferation rate. **b** Retroperitoneal sections from a mouse where NHL disease persists post-chemotherapy: similar histology of primary tumor is observed. **c** Liver sections from a mouse with complete remission: typical healthy hepatic architecture with the centrilobular veins and hepatic sinusoids is observed. **d** Tumor-infiltrated liver sections from a mouse where NHL disease persists post-chemotherapy: infiltrating tumor cells are observed in portal field. **e** Spleen section from mouse with complete remission: normal architecture with the *white* and *red* pulp is observed

A major aim of our work was to develop a lymphoma MRD model as a more suitable model for immunotherapy assessment. For this, we treated the animals with two doses of CHOP and achieved complete clinical remission during a period of around 20 days. To confirm complete remission in these animals, five mice per group were euthanized at day 45 p.t.i. to perform necropsies and histological analysis. Macroscopically, control animals (no treatment) showed disseminated disease with extensive lymph nodes and/or hepatic involvement (Fig. 3a). There were lymphoma infiltrations at inguinal and retroperitoneal lymph nodes. Instead, CHOP×2 animals did not show any disseminated disease (Fig. 3b). We also evaluated by histology the primary tumor site, draining lymph nodes, retroperitoneal lymph nodes, spleen, liver and other tissues of abnormal appearance in order to evaluate lymphoma infiltration. Hematoxylin-eosin stained sections demonstrated tumor tissue consisting of a diffuse growth pattern with large and cohesive tumor cells with pleomorphic nuclei resembling centroblast and some small/medium size centrocyte-like cells. Numerous small vessels (capillaries and arterioles) were visible among the tumor cells (Fig. 4). These cells were histological similar to human DLBCL. Liver involvement was 76 and 11% for control and CHOP×2 groups respectively as evaluated by histology. Liver lymphoma infiltration was characterized by atypical large lymphoid cells with central perivenous distribution within the liver parenchyma (Fig. 4d).

#### PET-CT evaluation

Advanced imaging techniques can improve disease assessment, not least because they allow response evaluation to treatment and follow-up without the need of sacrificing animals. We evaluated PET as an alternative for in vivo monitoring of the disease. For this, we first demonstrated glucose analogue (^18^F-FDG) uptake by A20 lymphoma cells in tumor-bearing mice confirming that ^18^F-FDG was a good radiotracer for PET imaging in A20 model.

**Fig. 5:**
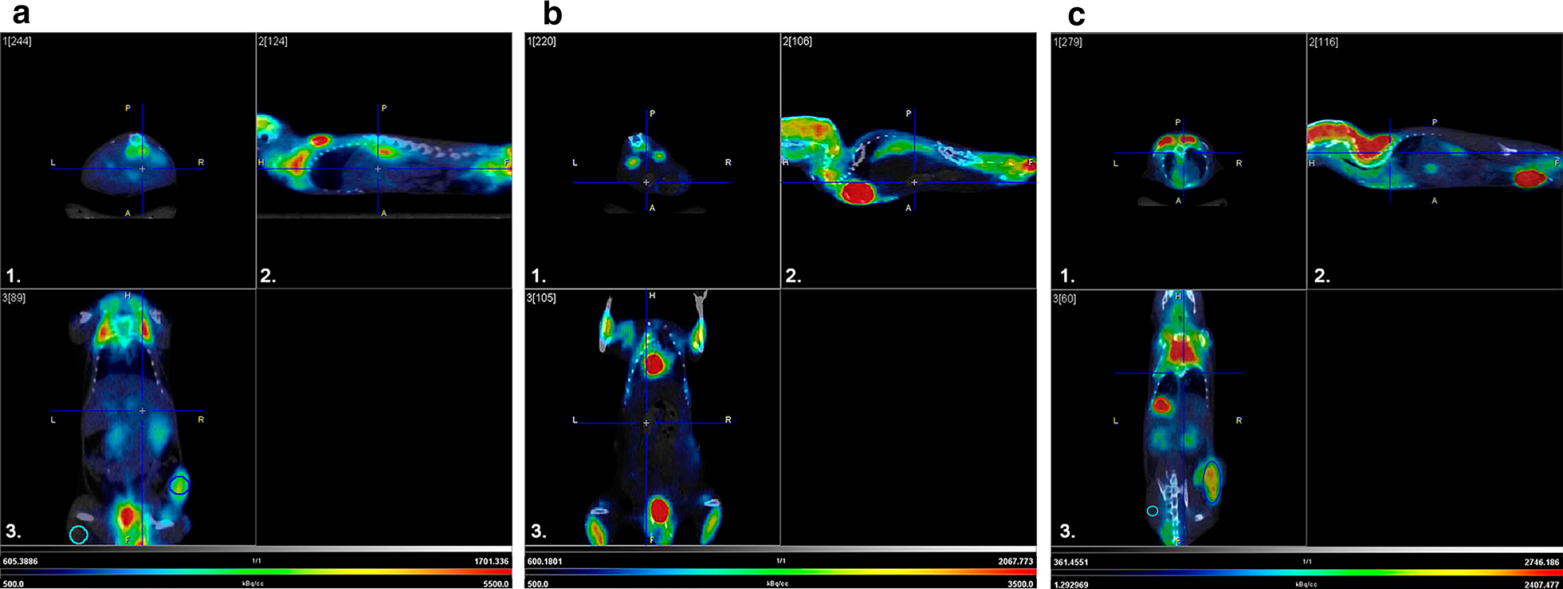
Positron emission tomography (PET) study of MRD. **a** A20 tumor-bearing mouse at day 25 p.t.i. (pre-CHOP treatment). Primary tumor FDG uptake is observed. Volumes of interest (VOI) are shown with *circles*: primary tumor (*blue*) and muscle (*skyblue*). T/NT: 4.5. **b** Animal with complete tumor remission at day 45 p.t.i. (post-CHOP treatment). FDG uptake is below detection level in primary tumor or in any organs. **c** Animal without tumor remission at day 45 p.t.i. (post-CHOP treatment). Primary tumor and hepatic metastasis are observed. VOIs are shown with *circles*: primary tumor (*blue*), hepatic metastasis (*red*) and muscle (*sky-blue*). T/NT: 4.0, liver/muscle ratio: 3.3. *1* axial section; *2* saggital section, and *3* coronal section

PET-CT imaging was performed before (day 25 p.t.i.) and after (day 45 p.t.i.) CHOP treatment in order to evaluate ^18^F-FDG uptake and to confirm the complete remission (MRD negative status). Post-chemotherapy, T/NT cut-off value was set to 2.45 and calculated using a ROC curve with histology as a gold standard. T/NT values of 2.45 or more were considered residual lymphoma disease. The AUC reached 0.74 (95% CI 0.6–0.991), with a sensitivity of 85.7% and specificity of 75%. Considering this cut-off value, pre-CHOP imaging (day 25 p.t.i.) indicated primary tumor uptake without metastasis as expected (Fig. 5a). On the other hand, post-CHOP PET image analysis (day 45 p.t.i.) revealed complete remission in many animals (Fig. 5b). Other mice had uptake at the site of the primary tumor (Fig. 5c), in disagreement with the histology result, suggesting the existence of false positive. Animals that had residual primary tumor after CHOP×2 treatment also presented retroperitoneum and liver ^18^F-FDG uptake (Fig. 5c) evidencing the existence of metastasis in accordance with histology results.

### Immunological status after chemotherapy treatment

**Fig. 6:**
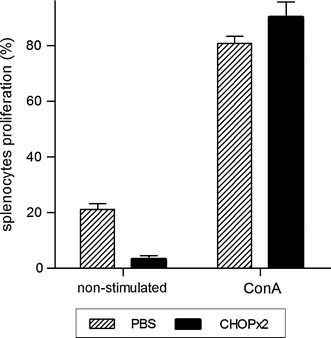
Splenocyte proliferation assay. Mice from PBS and CHOP×2 groups were sacrificed at day 45 p.t.i., and spleens were removed for splenocyte proliferation assay. Splenocytes were labeled with CFSE and cultured for 4 days with Concanavalin A (ConA) or left unstimulated. The percentage of proliferation cells for each condition was determined as described in M&M. Results are shown as the mean ± standard deviation (n = 5)

In order to assess the immunological status of CHOP-treated animals, five mice per group were sacrificed at day 45 p.t.i. and spleens were removed for proliferation assays. As shown in Fig. 6, mice in complete remission showed similar proliferation rate upon mitogen stimulation than control mice (PBS).

**Fig. 7:**
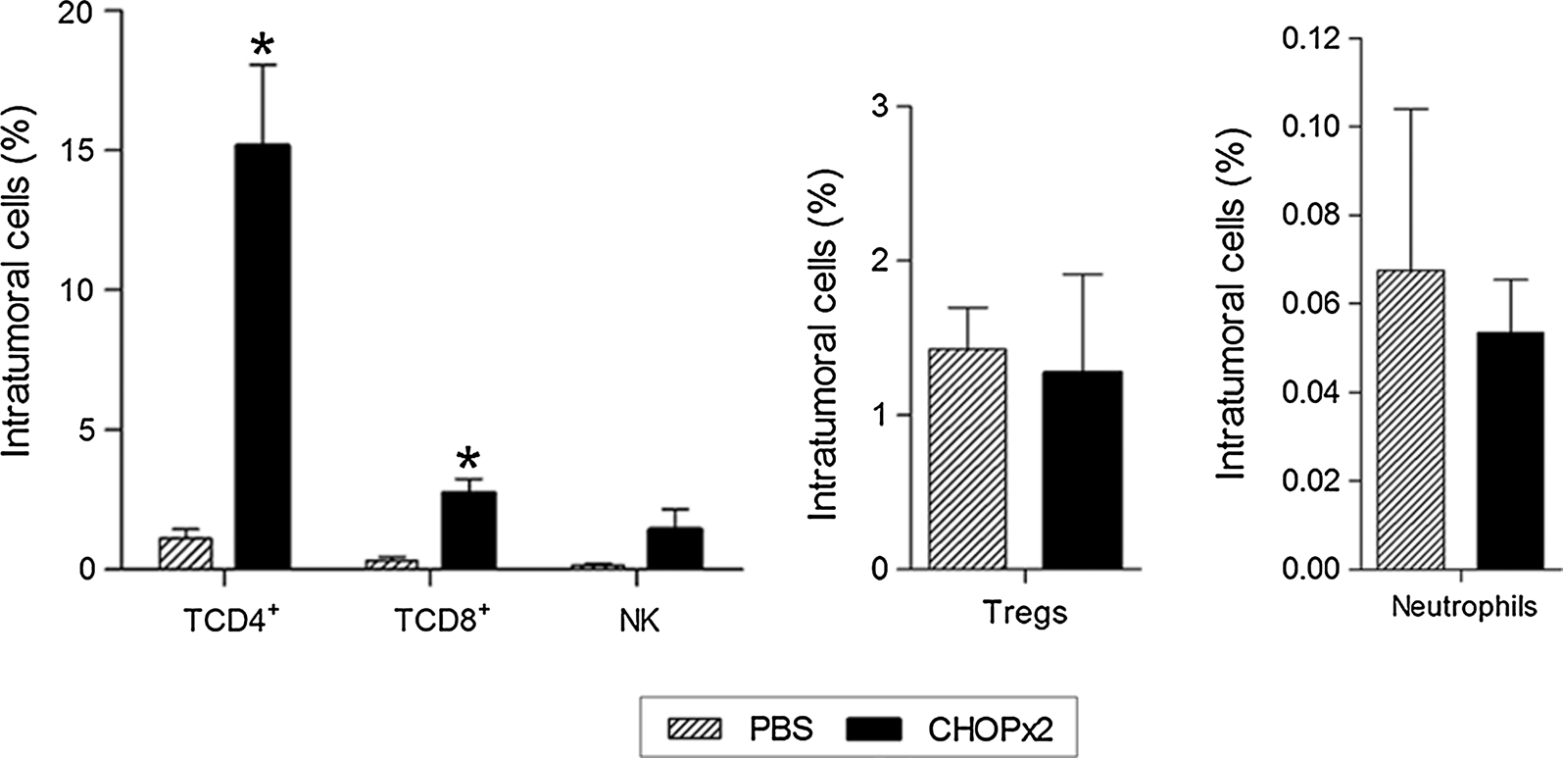
Tumor-infiltrating cells. Mice from PBS and CHOP×2 groups were sacrificed at day 45 p.t.i., and tumors were removed to study tumor-infiltrating cell populations. Percentage of CD4^+^ T cells (CD3^+^ CD4^+^ lymphocytes), CD8^+^ T cells (CD3^+^ CD8^+^ lymphocytes), NK cells (CD3^−^ CD49b^+^ lymphocytes), Tregs (CD3^+^ CD4^+^ CD25^+^ FoxP3^+^ lymphocytes), and neutrophils (Gr1^+^ CD11b^+^ cells), determined by flow cytometry. Results are shown as mean ± standard deviation (n = 5). Significant differences between groups were observed for CD4^+^ (p = 0.0023) and CD8^+^ T cells (p = 0.0034)

**Fig. 8:**
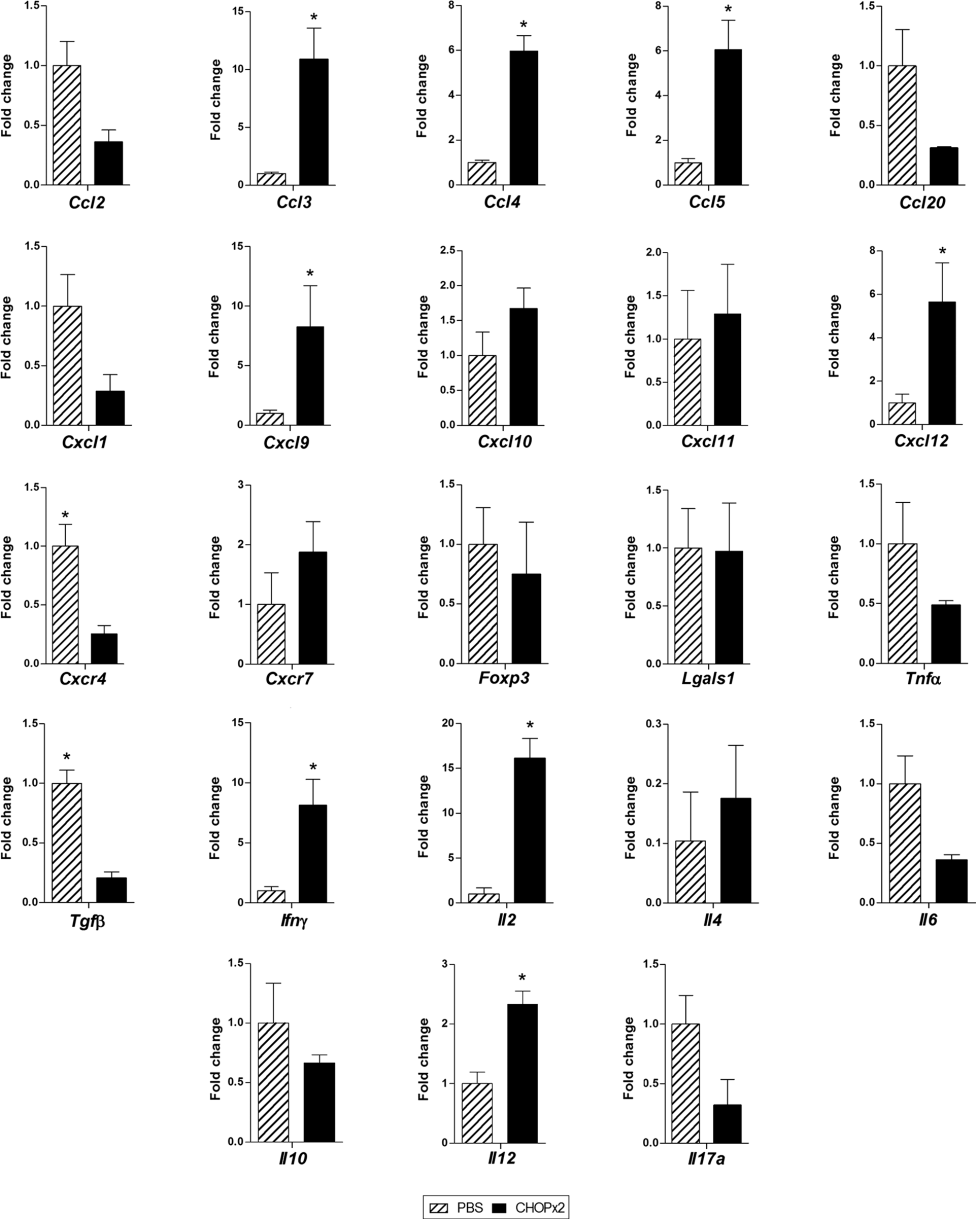
Cytokine/chemokine gene expression levels in the tumor microenvironment. Mice from PBS and CHOP×2 groups were sacrificed at day 45 p.t.i., and tumors were removed to assess the expression of cytokine and chemokines genes by quantitative RT-PCR on total tumor RNA. Gene mRNA values were normalized to that of *B2m* mRNA, and the results are expressed relative to mRNA levels in the PBS group. Results are shown as mean ± standard deviation (n = 5). Significant differences were observed for *Ccl3*, *Ccl4*, *Ccl5*, *Cxcr4*, *Cxcl9*, *Cxcl12*, *Il2*, *Il12*, *Ifng* and *Tgfb* genes (p < 0.05)

Additionally, mice with residual lymphoma (partial response) post CHOP×2 treatment were also sacrificed at day 45 p.t.i., and the primary tumor was removed to evaluate tumor-infiltrating populations. Animal from CHOP×2 group showed significantly higher numbers of CD4^+^ (p = 0.0023) and CD8^+^ (p = 0.0034) tumor-infiltrating T cells as compared with animals from the control group (Fig. 7). Numbers of NK cells, regulatory T cells (Treg) and neutrophils cells in the tumors were similar in treated and non-treated animals. Analysis of cytokines and chemokines mRNA levels in the tumor microenvironment of animals with partial response to chemotherapy showed significant increases in mRNA levels for *Ccl3*, *Ccl4*, *Ccl5*, *Cxcl9*, *Cxcl12*, *Il2*, *Il12* and *Ifng* whereas *Cxcr4 and Tgfb* gene expression were significantly repressed as compared with tumors from untreated mice (Fig. 8).

## Discussion

Therapies against NHL have achieved substantial success in the last few years [2, 3], but rates of relapse are still high fostering the research on new therapeutic approaches. Animal models are essential for research and development of novels immunotherapies, and the use of well-characterized inbreed laboratory mice is most likely one of the best experimental systems for cancer research. Cancer development has similar physiologic characteristics in mice and humans with the acquisition of mutations in equivalents oncogenes and suppression genes [20, 21]. Xenograft models are widely used to demonstrate pre-clinical activity of drugs prior clinical evaluation, but they are not adequate for testing immunotherapies or studying tumor microenvironment because these require immunocompetent mice [9]. Also, in order to more accurately predict human responses NHL immunotherapies should be tested in a similar context to that usually found in humans, which for most of the cases implies chemotherapy-treated patients. Chemotherapy can modulate distinct features of tumor immunobiology and the optimal integration of immunotherapies with standard chemotherapies to minimize antagonistic interactions and engage potential synergies is a major challenge [22]. Ideally, immunotherapy should be applied in the setting of MRD, after the tumor mass has been reduced with systemic chemotherapy. This strategy should minimize the negative impact of tumor bulk on the immune response and might work potentiating the immunological effects of chemotherapy. Indeed, the use of immunotherapy associated with traditional agents, as combination therapy, is now the standard for treatment of many types of cancer, improving clinical outcomes.

Many clinical trials with vaccines against NHL have shown anti-tumoral responses after achieving complete or partial remission with chemotherapy [3, 5, 23, 24]. For all these reasons, we propose that pre-clinical investigation in NHL should be attempted in a murine model that mimics that clinical setting.

The four-drug combination CHOP (cyclophosphamide, doxorubicin, vincristine, and steroids) with Rituximab has become the “standard” first line treatment for aggressive NHL (most frequently diffuse large B cell lymphoma) and it is the most used regimen for treat indolent lymphoma [1, 14, 25, 26]. In order to define more suitable pre-clinical conditions, we assessed the use of CHOP to generate a new model in A20-bearing animals that more closely resemble the clinical scenario. First, we tested the in vitro sensitivity of A20 cells to CHOP drugs. We found that the IC_50_ for doxorubicin and vincristine in A20 cells were similar to those reported by Dijoseph et al. in other lymphoma cell line [18]. Since cyclophosphamide and dexamethasone could not be evaluated because they require hepatic activation, we then tested the combination of drugs in vivo in A20-bearing animals, and found that a two-cycles drug schedule induces lymphoma remission for at least 20 days (MRD status) in a highly reproducible way. This schedule was well tolerated with transient and mild neutropenia and body weight loss.

We also found that administration of chemotherapy did not resulted in immunosuppression in A20-bearing animals. Cancer chemotherapy was historically considered as promoting immunosuppression, but recent studies have demonstrated that certain chemotherapies can instead augment tumor immunity in addition to their cytotoxic effect. Chemotherapy can promote anti-tumor immune responses by increasing the immunogenicity of malignant cell, or by inhibiting immunosuppressive circuits that are established by cancer [27–29]. Some chemotherapy drugs at their standard dose mediate their tumor effect, at least in part, by inducing immunogenic cell death, which involves the release of tumor antigens and danger-associated molecular patterns (DAMPs) in the tumor microenvironment, resulting in a proinflammatory microenvironment, where specific anti-tumor immunity may be developed [28]. It has also been demonstrated that chemotherapy can modulate the activity of immune T-cell subsets decreasing the number of Tregs cells, shifting CD4^+^ T helper phenotype from type 2 to type 1, inducing the differentiation of Th17 cells, and promoting the evolution of a durable T-cell memory [28]. We found that animals receiving the two CHOP doses (CHOP×2 group) have higher numbers of CD4^+^ and CD8^+^ T cells recruited to the tumor as well as a significant increase in *Il12* and *Ifng* and a decrease in *Tgfb* gene expression reinforcing the idea that CHOP treatment may indeed stimulate the development of effective antitumor immunity [30].

Increasing evidence indicates that the tumor microenvironment has critical roles in all aspects of cancer biology, including growth, angiogenesis, metastasis and progression. In this regard, it is recognized that chemokines and their receptors, originally identified as mediators of inflammatory diseases, serve as critical communication bridges between tumor cells and stromal cells to create a permissive microenvironment for tumor growth and metastasis [31–34]. In melanoma, it has been demonstrated that up regulation of expression of several chemokine genes such as *Ccl2, Ccl3, Ccl4, Ccl5, Cxcl9, and Cxcl10* in the tumor microenvironment correlates with the recruitment of activated effector T cells to the tumor increasing antitumor immunity [35]. Conversely, it has also been reported low amount of these chemokines in poorly infiltrated melanomas and colorectal carcinomas [35–37]. Our results showing a significant increase in *Ccl3, Ccl4, Ccl5* and *Cxcl9* genes expression in CHOPx2 group versus PBS group at day 45 p.t.i. are in accordance with those previously reported results, and confirm that in our model post-chemotherapy mice are fully immunocompetent. Furthermore, in CHOP×2 group we found a significant increase in *Cxcl12* expression but not in expression of its receptor *cxcr4*. The CXCL12–CXCR4 biological axis has been associated with tumor invasion and metastases. In fact, CXCR4 is the most common chemokine receptor overexpressed in human cancer [32].

To confirm remission and monitored the disease an in vivo imaging system (PET) was applied in our model. When tumor cannot be detected clinically the use of PET post-chemotherapy allows a diagnosis of complete remission. PET is a minimal invasive technology easy to perform on anaesthetized mice, offering serial dynamic observations of tumor development in the same animal. In recent years, the use of PET in the diagnosis and monitoring of NHL patients, as well as other types of cancer, has increased [30, 38]. The most frequent radiotracer used in oncology is the glucose analogue FDG labelled with the positron-emitting radionuclide ^18^F. Several studies have demonstrated ^18^F-FDG increased uptake in most lymphomas, with a good correlation between intensity uptake, malignancy and proliferation activity [39]. Chaise et al. demonstrated the feasibility of PET using ^18^F-FDG in high-tumor burden A20 murine model [40]. In this study we could evaluate the treatment response detecting intra-abdominal lymphoma and hepatic involvement. There was a good correlation between PET images and histology. To the best of our knowledge, our study is the first to assess the value of FDG-PET for in vivo evaluation of post-chemotherapy remission in a murine NHL model. In patients, it is accepted that post-chemotherapy inflammatory reactions increase the rate of false positives results obtained with PET. To minimized this in NHL patient the PET scan should be performed after at least 3 weeks post-chemotherapy [41]. A meta-analysis performed by Zijlstra et al. revealed a pooled sensitivity and specificity of 72% (95% CI 61–82%) and 100% (95% CI 97–100%), respectively [42]. In our PET studies we reached a good sensitivity (85.7%) whereas specificity was 75%. The lower specificity results compared with data reported in human could be explained by the remaining inflammation at the time of our analysis.

Overall, we describe a NHL-B MRD syngeneic murine model as a novel tool to evaluate immunotherapies effectiveness as well as to study tumor microenvironment in relapse lymphoma. This model is unique in many aspects, inasmuch: (a) it uses fully immunocompetent mice; (b) remission is obtained by CHOP chemotherapy using the same cytostatic drugs that are the first line standard treatment for NHL in humans; (c) lymphoma complete remission is reached and maintained for a reasonable therapeutic window (20 days) where immunotherapies can be tested; (d) animals remains immunocompetent despite chemotherapy; (e) it uses an in vivo imaging system to follow disease evolution that correlates well with histology as gold standard.

## Conclusions

We have developed a novel pre-clinical model for NHL lymphoma that resembles more closely the situation found in humans, providing a therapeutic window where immunotherapies can be tested in conditions closer to those found in patients. We feel that the availability of such a model provides an important tool for evaluating new immunotherapies and will help to bridge the gap between pre-clinical models and clinical trials.

## Abbreviations

ANOVA: analysis of variance
AUC: area under the curve
BWC: body weight change
CFSE: 5(6)-carboxyfluorescein diacetate *N*-succinimidyl ester
CHOP: cyclophosphamide, doxorubicin, vincristine, steroids
CHOP×1: one cycle of CHOP regimen
CHOP×2: two cycles of CHOP regimen
ConA: concanavalin A
CRT: calreticulin
CSR: cancer statistics review
DAMPs: danger-associated molecular patterns
DLBCL: diffuse large B cell lymphoma
3D-MLEM: 3D maximum likelihood expectation maximization
DMSO: dimethyl sulfoxide
^18^F-FDG: ^18^F-2-Fluor-2-desoxi-d-glucosa
IC_50_: half maximal inhibitory concentration
i.p: intraperitoneal
IPI: international prognostic index
MRD: minimal residual disease
NHL: non-Hodgkin lymphoma
NT: non-target tissue
PBS: phosphate-buffered saline
PET/CT: positron emission tomography/computed tomography
PI: propidium iodide
p.t.i: post-tumor implantation
ROC: receiver operating characteristic
RT-PCR: real time-polymerase chain reaction
s.c: subcutaneously
SEER: surveillance, epidemiology, and end results program
T: target tissue
Tregs: regulatory T cells
VOIs: volumes of interest
